# Algorithm for Designing a Removable Complete Denture (RCD) Based on the FEM Analysis of Its Service Life

**DOI:** 10.3390/ma15207246

**Published:** 2022-10-17

**Authors:** Dmitry I. Grachev, Nurmukhamet S. Ruzuddinov, Anatoliy S. Arutyunov, Gadzhi D. Akhmedov, Lubov V. Dubova, Yaser N. Kharakh, Sergey V. Panin, Sergey D. Arutyunov

**Affiliations:** 1 Digital Dentistry Department, A.I. Yevdokimov Moscow State University of Medicine and Dentistry, 127473 Moscow, Russia; 2 Department of Clinical Specialties, Al Farabi Kazakh National University, Almaty 050040, Kazakhstan; 3 Propaedeutics of Prosthetics Technologies in Dentistry Department, A.I. Yevdokimov Moscow State University of Medicine and Dentistry, 127473 Moscow, Russia; 4 Propaedeutics in Surgical Dentistry, A.I. Yevdokimov Moscow State University of Medicine and Dentistry, 127473 Moscow, Russia; 5 Orthopedic Dentistry Department, A.I. Yevdokimov Moscow State University of Medicine and Dentistry, 127473 Moscow, Russia; 6 Laboratory of Mechanics of Polymer Composite Materials, Institute of Strength Physics and Materials Science of Siberian Branch of Russian Academy of Sciences, 634055 Tomsk, Russia

**Keywords:** removable complete denture (RCD), clinical reasoning, dental restoration failure, dental stress analysis, denture bases, denture design, finite element analysis, jaw, edentulous, prosthodontics, tooth, artificial

## Abstract

(1) Background: The paper addresses the computer simulation and prediction of the service life of the base of removable complete dentures (RCDs) under typical loads caused by biting and chewing food. For this purpose, the finite element method (FEM) was used. It is assumed that various blocks of teeth, such as incisors, canines, premolars and molars, are subjected to cyclic impacts during a human life. (2) Methods: Both symmetric and asymmetric mastication (two- and one-sided loads, respectively) cases were considered. The load level was assumed to be 100 N, which corresponds to the average muscular compression force of typical human jaws. (3) Results: The FEM analysis of the stress–strain state evolution for RCDs under cyclic loads was carried out. Maps of equivalent lines were drawn for the denture base in terms of its durability. A multi-axial criterion was implemented to determine the number of cycles prior to failure by the mechanism of a normal opening mode crack. The FEM-based assessment of the service life of RCDs enabled us to establish the critical stress concentration areas, thereby allowing for further planning for the correction of an occlusal scheme or teeth inclinations. As a result, the service life of RCDs under cyclic loading can be improved. (4) Conclusions: An algorithm for designing RCDs in the case of edentulism based on the FEM simulation using commercial software as part of the procedure is proposed.

## 1. Introduction

The use of removable complete dentures (RCDs) is a common method for prosthodontic treatment, especially for elderly patients [1,2]. There is a significant variety of suitable structural materials, designs and treatment strategies, the implementation of which depends on the anatomical specificity and the response of each patient to the fitting of an individual denture [3,4,5]. The experience of using RCDs has been constantly summarized, which constitutes the basis for the development of appropriate practical recommendations [6,7]; however, some challenges in dental treatment procedures, as well as the development of new computer simulation and manufacturing methods, have attracted great attention from researchers and practitioners [8,9,10]. In 2020, in the United States alone, the number of patients that needed complete denture treatment was estimated to be about 61 million people [11].

According to both anatomical and functional purposes, the dentitions of both jaws are divided into functionally oriented groups. The front one consists of eight incisors for biting food and four canines for the separation of its pieces, which are associated with the application of great effort (load). They are followed by eight premolars for the capturing and subsequent grinding of food. The structure of the premolar is almost identical to the canines. Posterior teeth possess a prismatic shape and an expanded surface. Behind the premolars, 12 molars are located for the grinding of food under significant cyclic loads. These teeth have a crown shape with a large mastication surface, upon which four to five cusps are present (three in the front and two behind) [12]. Unlike natural individually fixed teeth, an artificial dentition is attached as a single unit on a polymer base.

Between opposing teeth, great loads develop during occlusion (from 10 up to 3000 N, according to various references). Moreover, a complete denture experiences multidirectional cyclic loads during conversation and mastication (which also depend on its consistency). Additionally, possible errors in the occlusion design cause instability in the prosthesis structure during edentulism. The quality of manufactured RCDs is greatly correlated with the satisfaction of the prosthodontic-treated patients and the quality of their life [13,14,15].

Several reasons may result in a breakage of such prostheses, which include:

- some mistakes by a dentist (in designing a plaster cast of the supporting tissues and/or shortening the boundaries of the denture base around the perimeter; and in the positioning of the mandible in relation to the maxilla, as well as in determining the height of the mandible);
- any technical faults by a dental technician when arranging the artificial teeth and a denture base;
- the breaching plastic polymerization procedures;
- shortening the denture base boundaries upon grinding and polishing;
- the pronounced atrophy of the alveolar ridge in both jaws;
- a lack of congruence between the denture base and supporting tissues;
- inappropriate physical, chemical and mechanical properties of applied structural materials;
- inaccurate use of the prosthesis by a patient (falling, damaging, etc., when removing or inserting).

Many results from both case studies and systematic investigations on the reasons for failure of RCDs have been published in medical papers, the list of which is far from limited to the following references [16,17,18,19,20,21,22]. The authors of the current research are familiar with various manifestations of this issue not only from the reported data, but also from their own experience in designing and applying prosthodontic treatment to edentulous patients [23,24,25]. The multi-year background of patients that used complete dentures was the basis for the development of practical recommendations for dental technicians and prosthodontists, which are represented in the form of the algorithm in the final part of this paper. Its goal is to report the principles of how to design RCDs, with a focus on ensuring their long-term and comfortable use by a patient.

Before discussing the algorithm for designing the RCDs, the authors considered the list of reasons that would additionally determine the relevance of the development of such an approach, among which:

- the specifics of the interaction between an alveolar ridge and the RCD base [26,27,28,29];
- the influence of different inclination angles of the printed artificial teeth on the denture base [30,31,32,33,34];
- the need to adjust the treatment strategy by taking into account the dentures’ design [35,36,37,38,39];
- some individual characteristics of the patients in terms of their denture exploitation and mastication [40,41,42].

**Figure 1:**
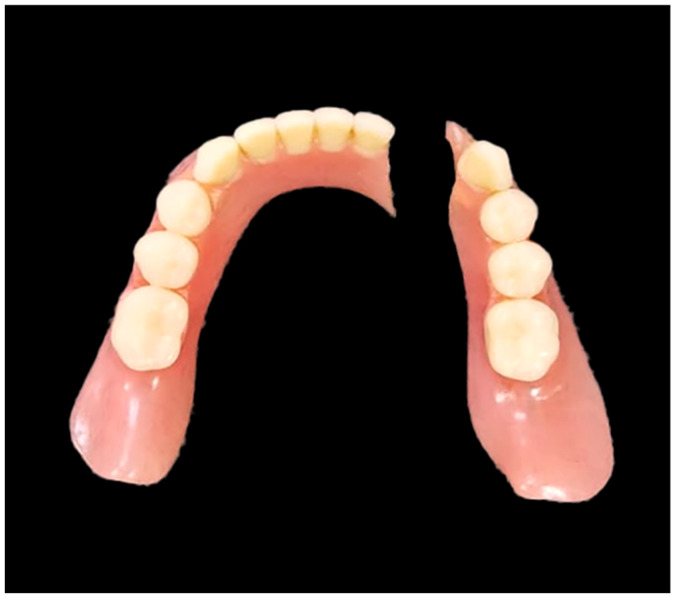
An example of a fractured acryl denture base.

Despite the existing occlusal schemes, the actual result from arranging the artificial teeth is associated with additional factors, such as the anatomical features of the oral cavity and/or aesthetic preferences of a patient [43,44]—since the teeth can be displaced in the vestibular or oral directions. These displacements/inclinations determine the occurrence of stress concentrators, thereby causing the possibility for a fatigue fracture. An analysis of the results of both our studies and the published data has enabled us to conclude that the vestibular displacement of the front teeth blocks can give rise to a 2.5–3.0-times increase in local stresses, while an increase of up to 4.0 times can be observed for the posterior [45]. The main crack initiation and propagation leads to denture failure (Figure 1). Thus, the displacement of the functionally oriented blocks of the artificial teeth or their inclination can multiple, thereby varying the service life of RCDs.

The anatomical and physiological characteristics of each patient are unique. Moreover, they can change while using dentures. This requires a deviation from the classic elements of dentition design, for example, via the additional reinforcement or modification of the configuration of the denture base. An effective way to solve this problem can be through the implementation of computer simulation algorithms, primarily the finite element method (FEM) [46].

Due to the specifics of the structure and interaction of both RCDs and supporting tissues [47], the FEM analysis remains the most attractive method [48]. It was shown that the Von Mises stress patterns possessed unique variations and regularities within the model, given the consideration of various posterior occlusion types. In addition, higher stresses were found at a cuspal angulation of 33° and 20° in comparison with a 0° inclination.

Since RCDs are a component fixed on a supporting structure, it was shown in [34] that the arrangement of the posterior artificial teeth should be analyzed for their denture stability and avoidance of high pressures on the alveolar ridge. In doing so, the mutual displacements and elastic interactions of the alveolar ridge and the (mandibular) elastic complete denture should be taken into account [49].

In [50], the fatigue behavior of the complete denture, which is fabricated from the ‘SUPERACRYL PLUS’ material (Spofadental a.s., Jicin, Czech Republic), was assessed with the ‘ANSYS’ FEM-based commercial software package. In this case, Weller’s curves were drawn for the flat samples. The fatigue tests of the RCDs were conducted with a wood-based composite material in order to vary the applied loads. The simulation results were presented in terms of the safety factor variation. The authors of [50] suggested implementing a FEM-based procedure for eliminating the total damage (accidents), which is a characteristic of complete dentures.

In order to select the materials and design of RCD structures, an assessment of the developing stresses is required. In particular, the relationship between the alveolar ridge and the distribution of stresses on the mucous membrane should be determined. In addition, the contact between the denture base surface and the mucous membrane should be evaluated [51]. It was shown by the FEM analysis that the maximum stresses were localized in the alveolar ridge with a severe absorption, while the distal load was characterized by great compression stresses on the inclined plane of the rear alveolar ridge.

In addition to the FEM analysis, which is widely implemented for solving problems in order to calculate the stress–strain states (SSS) of RCDs considering the complexity of their shapes and surfaces, the boundary element method was proposed in [52]. This method allows us to obtain more accurate calculations (in contrast with the FEM ones) in some cases.

The authors consider it important to cite some papers in the field of the FEM analysis of complete dentures. These papers highlight: (1) the wide application of this approach for solving practically urgent problems, (2) the breadth of the applied models and criteria, and (3) the absence of universal rules for their application [12,32,53,54].

In the present paper, the authors dealt with RCDs for edentulous patients. Its fastening does not involve the installation of fixing pins—unlike the use of implants [55,56,57,58]. The aspects of the mechanical behavior of such dentures are relevant as they enable us to correct the design and take into account the particular features of each patient.

The engineering calculations of the dental prosthesis’s strength are mostly based on studying their SSS under static loads. The applied approach enables us to assess the strain fields under maximum static loads and their corresponding stresses, which can be compared with the material mechanical properties, such as the yield point or the ultimate tensile strength, depending on some hypothetical preconditions.

Nevertheless, a failure in one half-cycle of loading is generally not a characteristic of RCDs, except in cases where serious material discontinuities or other fatal factors are present. The most typical failure mechanism during the operation of complete dentures is gradual damage accumulation under cyclic loads and the formation of a fatigue crack. Thus, there is a need to develop methods for estimating the service life of such structures based on computer simulation results.

In continuum mechanics, multi-axial fatigue strength criteria are implemented to assess the safe life of the structural elements that are being subjected to cyclic loading. There are two broad classes of such criteria, which include the equivalent strain and stress values. The choice of one or another type is determined by the magnitude of the external functional loads. For example, if cyclic loads cause significantly non-linear strains with an accumulation of residual or plastic deformations, then the strain-based criterion is applied to determine the service life of a component in such a loading mode; if only elastic strain occurs under functional loads, then the stress criterion is used. In addition, there are uni- and multi-axial strength criteria depending on the load pattern and the resulting SSS.

If the SSS is quasi-homogeneous, then the uni-axial criterion is applied. For the significantly inhomogeneous SSS, a combination of the stress–strain tensor components is adopted to assess the strength of a component. The basic hypotheses about the most dangerous loads determine the choice of the appropriate criterion. For example, individual plates are weakly bonded with a cementing material in the case of a layered medium, so the hypothesis of the shear failure mechanism is applicable. An additional factor may be a simultaneously applied tensile load across the layers. In this case, the criterion contains the stress tensor components that describe the shear strains, as well as the ones that correspond to a normal opening.

The fatigue strength criteria establish a correspondence between the stress magnitude and the maximum number of cycles prior to a failure. In the first approximation, the analysis is carried out at a given external load, which in reality does not correspond to any actual conditions; however, a lower bound estimation of the product’s durability is obtained if the maximum possible load is applied. In other words, if a patient clenches his/her jaws with maximum effort, then the prosthesis durability has to be no less than the calculated period. In everyday life, mastication at maximum loads is not common. This fact is evidence of a greater durability level compared to the predicted one. An analysis based on the maximum values of the functional loads ensures that safe complete dentures are being designed.

Thereby, the multi-axial criterion of the static or cyclic strength is the most common one in the case of a structure durability analysis—just as a rule. This paper reports on the analysis of some potential fracture areas for RCDs under typical loads, and suggests a methodology for the service life assessment (i.e., the number of cycles before a failure) of RCDs designed for mandible edentulous patients.

## 2. Materials and Methods

The developed model includes the artificial teeth and a denture base. These two elements of RCDs are made from materials with different mechanical properties (Table 1). The dentition material was chosen to be harder and more durable. Bonding of the dentition and the foundation was carried out with a polymeric material, which was accompanied by the formation of a chemical bond. For this reason, the ideal adhesion condition was specified in the model.

In order to study the SSS of the base of a complete denture of RCDs, which are used as prosthetics for edentulous patients—as well as to assess its service life—a three-dimensional model was developed. The latter simulates the mastication-induced loads and consists of a geometric Computer Aided Design (CAD) model and a physical contact interaction one.

### 2.1. The Geometric CAD Model

**Figure 2:**
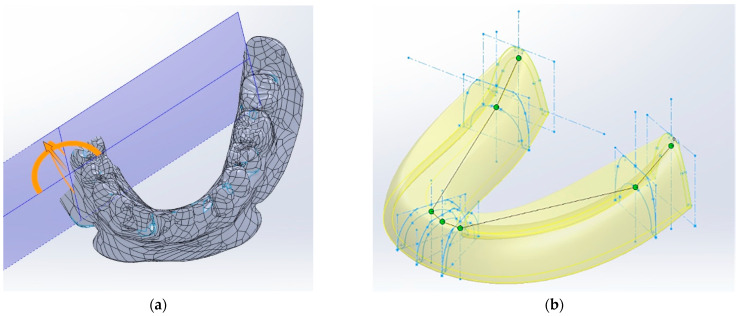
Images of the base of the RCDs obtained with a 3D scanner (**a**) and the three-dimensional model designed in SolidWorks software (**b**).

When constructing the geometric CAD model, the characteristic dimensions of a mandible denture for a 50-year-old male patient were utilized. They are quite comparable with the ones used in [12]. A 3D scan of the RCDs was chosen as the reference data (Figure 2a). Using a virtual STL model in the SolidWorks software, the reference sections of the denture base were designed (Figure 2b). Then, the three-dimensional model was formed using the profile wrapping tool. Some extra approximating surfaces were formed in advance, along which both boundary conditions for fixing the denture on the mandible and the surfaces for applying functional loads were set. The functional loads were transferred to the occlusal surface of the corresponding tooth blocks.

### 2.2. The Physical Model of the Denture Base of the RCDs

The physical model describes the main contact interactions, as well as both the physical and mathematical designations of the relationships of the material’s behavior. In addition, this justifies the initial and boundary conditions. The key objective of the study was to obtain computer simulation data on the types of stresses observed in the denture base under typical loads. Their levels were determined by the state of the masticatory system of a person and can reach several hundred Newtons.

Based on an analysis of the maximum load levels, the indicated problem could be considered within the framework of the linear theory of elasticity, and the behavior of the materials could be assumed to be completely elastic. The materials of the denture base and the dentition were implied to be homogeneous and isotropic. The computer simulations were performed by anticipating a constant temperature without taking into account any of the effects associated with eating hot or cold food. Within the framework of these assumptions, built-in algorithms and methods for the numerical solving of linear elasticity approximations used in the FEM-based SolidWorks 2010 software package (Dassault Systems, France) were applied. Tetrahedral elements with ten nodes were used. Both the shapes and sizes of the finite elements were chosen on the basis of their variation and for the study of the convergence of the obtained results.

### 2.3. The Boundary Conditions

RCDs have a large contact surface with a mucous membrane of supporting tissues. When developing a physical model of the denture base, it was necessary to pay special attention to the biomechanical behavior of the mucous membrane. Different parts of the latter are characterized by various compliances. The minimum-level regions were identified as the greatest contributors to the compensation of the functional loads on the RCDs base. The physical model of the mucosa for these regions is based on the assumption that the mechanical properties of soft tissues change with an increase in the applied force. This process can be explained by a decrease in the saturation of blood vessels in tissues that are subject to external compressive forces.

When the fluid-containing component is removed from the contact area, the mechanical system’s compliance is sharply reduced. Mathematically, this means that the mucosal compliance is a function of the compressive stresses. For the simplest case, a linear relationship can be used, which can relate the mucosal compliance value in the unloaded state and can relate when the pain threshold for the patient is reached. In this research, a linear dependence of the soft tissue compliance on the normal displacement of the surface of the RCDs base, when in contact with the mucosa, was chosen without a loss of generality.

**Figure 3:**
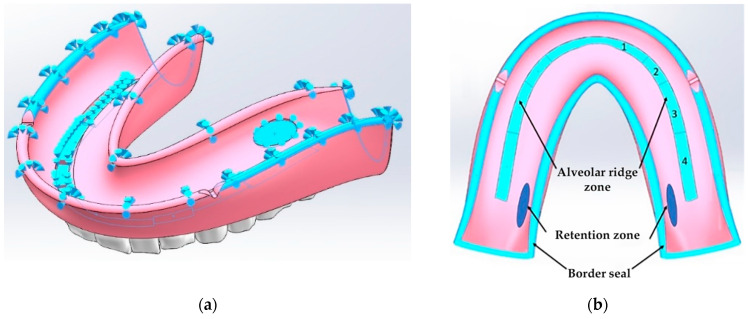
Schemes of the base of the mandible dentures with marked contact surfaces (**a**) and areas of minimal mucosal compliance (**b**).

The considered characteristic areas with a minimal and linearly changing compliance differ for the denture base of the maxilla and mandible. For the upper one, the minimum compliance values are observed in the area of the torus, while in the second case, they are at the top of the alveolar ridge (Figure 3).

In the present study, the structural integrity was investigated and the durability of the base of the mandible RCDs was assessed. As noted above, the area with a minimal mucosal compliance is located at the top of the alveolar ridge for this configuration. To ensure the structural stability under the uniform loading of the denture base (the same pressure applied to all blocks of teeth), a segmented area of the minimal mucosal compliance was chosen.

Four segments were allocated on each side of the RCDs base relative to the axis of symmetry. The compliance of each of the segments was selected so as to exclude any inclinations of the denture base in both the oral–vestibular and frontal–rear directions. As additional fixing areas, the following ones were distinguished: (1) a border seal located along the perimeter of the base (Figure 2b) and (2) the zones in which the forces, which are similar in their pattern to the friction ones, can become apparent due to the base shrinkage. In Figure 2b, these elliptical regions are indicated in darker colors at the back of the RCDs base (the retention zones). In these fixing regions, the forces that arise are directed opposite to the local displacements. As a result, the physical model of the boundary conditions included blocks, which describe: (1) the mucosal; (2) the alveolar ridge; (3) the border seal; and (4) the retention zones.

### 2.4. The Loading Conditions

In order to develop the closed physical model, it was necessary:

(1) to determine the elastic and strength properties of the structural elements;
(2) to set the boundary kinematic or dynamic loads;
(3) to set constraints that ensure the structure’s balance.

The stiffness levels (E_basis_ = 1000 MPa, E_tooth_ = 2000 MPa) were defined according to the material specifications (Table 1). The ultimate compressive strength of the RCDs base material was assumed to be 60 MPa. Upon mastication, the load level was 100 N, which corresponds to the average muscular compression force of a typical human jaw. The applied pressure in each individual case was determined by the surface area of the loaded teeth

$$S_{k}:\;P_{k}=\frac{F_{0}}{S_{k}}$$

**Figure 4:**
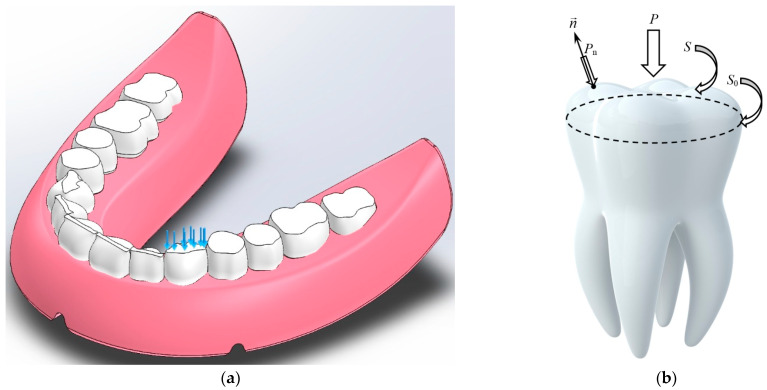
A model of the base of the complete denture with the artificial teeth (dentition) (**a**) and a schematic representation of a tooth with the loads applied (**b**).

The research considered the cases of applying the load to individual blocks of teeth. Depending on the mastication surface area, the load levels varied from 1 MPa up to 10 MPa. Figure 4 shows a case wherein the entire load was concentrated on the surface of a canine, while all other teeth were unloaded.

The tooth loading scheme should be considered with a distributed vertical force *P*. In this case, the tooth mastication surface possessed an arbitrarily curvilinear shape with an area equal to *S*, while the surface projection area on the horizontal plane was equal to *S*_0_ (Figure 3b), respectively,

$$S=\int_{\Sigma }dS,\;S_{0}=\int_{\Sigma }\sin\beta \left(x,y,z\right)dS$$

where β is the normal angle of the horizontal plane at a given point on the surface, and sinβ⋅dS is a surface element projection onto the horizontal plane. By comparing the load on a real tooth with that on another one, the flat mastication surface *S*_0_ was characterized and the following expression was obtained:

$$F_{0}=P\cdot S_{0}=P\cdot \int_{\Sigma }\sin\beta \left(x,y,z\right)dS$$

At each point of the real surface, the normal force component acts as:

$$P_{n}=P\cdot \sin\beta \left(x,y,z\right)$$

Consequently,

$$F=\int_{\Sigma }P_{n}dS=P\cdot \int_{\Sigma }\sin\beta \left(x,y,z\right)dS=F_{0}$$

As a result, the same integral force acts on the flat mastication surface as it does on the real tooth with the same vertical load. In fact, RCDs press on the surface of a part of the alveolar ridges at the mandible (or at the maxilla). Since the palate, as well as any parts of the alveolar ridge, was not taken into account in the developed model, their impact on the denture base was considered using the “elastic foundation” option. The elastic foundation rigidity was selected from the equilibrium conditions of the dentures model. Since the rigidity of a bone is much higher than that of the mucosa and the RCDs base material, the normal displacements of the latter were assumed to be zero where there was contact with the torus.

**Figure 5:**
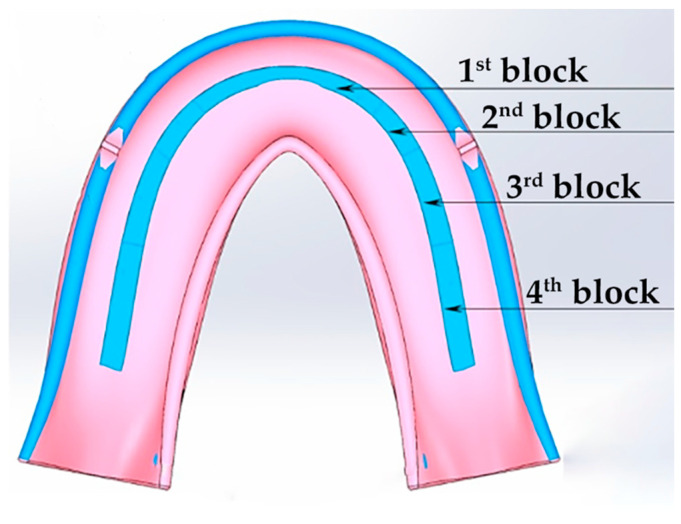
The schematic representation of the boundary conditions on the contact surface of the dental base of mandible RCDs.

Several gingival contact zones were identified for the mandible (Figure 5). For each, their own value of the base stiffness could be set. By varying the stiffness of the virtual denture base, any constraint degrees could be simulated (from weak backwater to full fixation).

**Figure 6:**
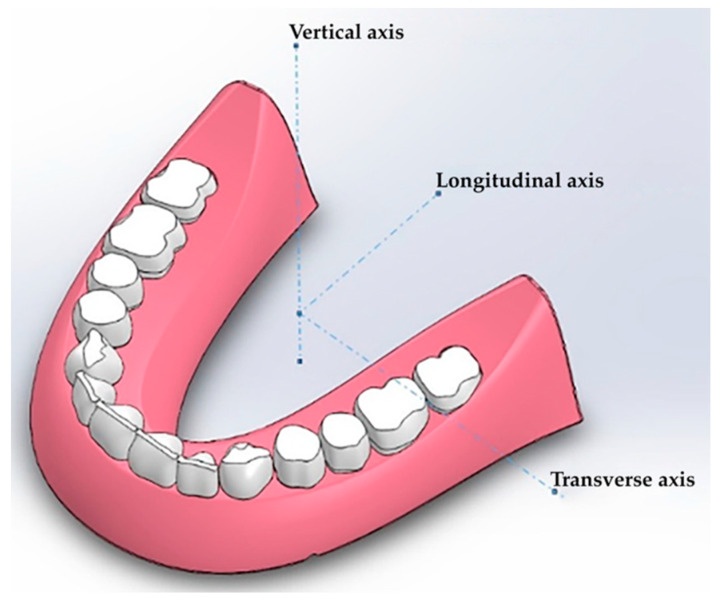
The scheme of the potential rotation axes around the applied force center.

Since the denture design does not provide any strict kinematic restrictions (fixed displacements), the problem of its rotation around the center of the applied forces may arise. Three rotation components are possible (Figure 6), namely around the longitudinal, transverse and vertical axes (the roll, pitch and yaw angles, respectively).

**Figure 7:**
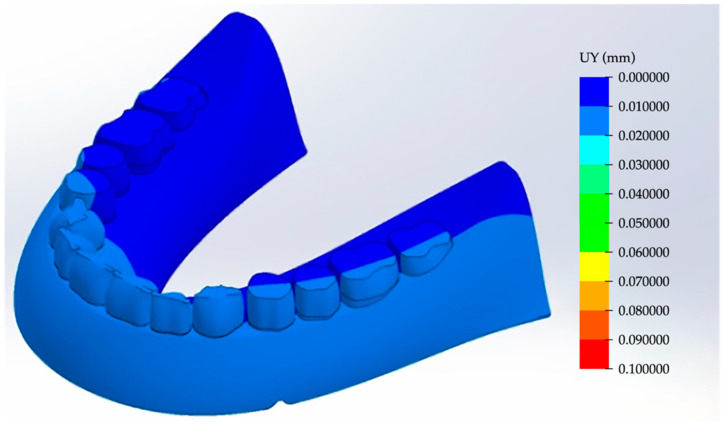
The schematic representation of the load distribution on the mandible RCDs base.

The load does not have any horizontal component, so there is no significant yaw. Both the pitch and roll angles arise due to the asymmetrical load on a tooth. In this case, a “background” rotation takes place, which does not allow for the adequate analysis of the the prosthesis strain—if the restrictions are not balanced. Therefore, it was necessary to solve the problem by pre-setting the stiffness of the virtual RCD base, at which point the pitch and roll angles were vanishingly negligible for the symmetrical load over the entire surface. The load was then evenly distributed over all teeth, as shown in Figure 7.

In this case, the component was almost balanced. The pitch and roll were practically absent, and the movement range was within 0.02 mm. Accordingly, it could be stated that the development of the pitch and roll were a direct result from the load asymmetry or the teeth arrangement while maintaining the balance in the calculations for such loading constraints. This enabled an adequate analysis of the effects of the SSS redistribution, thereby revealing the characteristic features of the considered loading method.

## 3. Results

### 3.1. Static Loading

The equivalent stresses (the Mises criterion) were used for analyzing the strain behavior of the RCD. Along the principal axes of the stress tensor, there were a combination of its principal components. This criterion determined the beginning of the plastic flow process when the equivalent loads reached the material yield point. For this reason, the areas with maximum values for equivalent stresses could be characterized as potentially dangerous for the loading case that was under consideration. It should be noted that such conditions do not mean onset of the critical state. The onset of the fracture in the detected region can be established only after comparing the levels of the equivalent stresses with the mechanical properties of the material.

**Figure 8:**
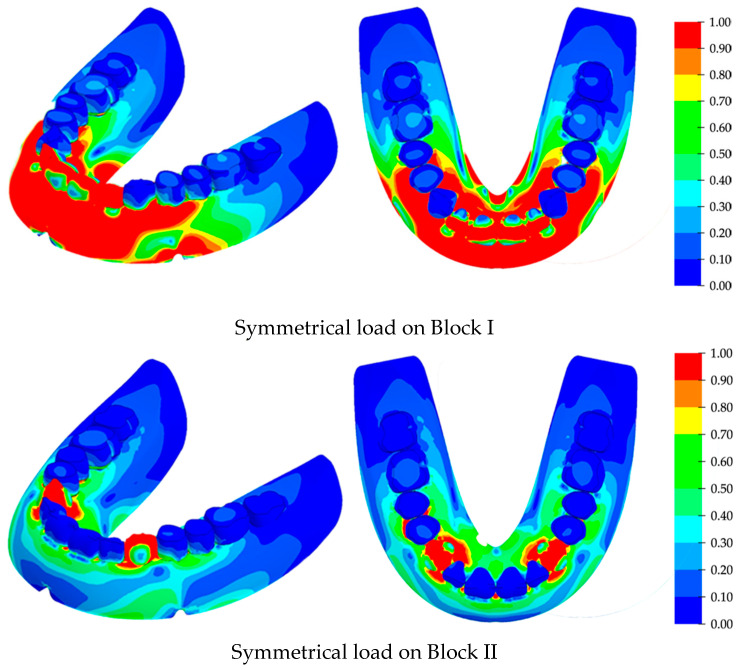
The distributions of the maximum stresses under symmetrical loads on various blocks of teeth: isometry on the left; the bottom view on the right.

The computer simulation results are presented for the case of the direct arrangement of teeth along the crest of the alveolar ridge under both symmetric and asymmetric loads. The calculation was performed for the cases of sequential loading of the blocks of tooth from the first to the fourth. For all these loading configurations, the most dangerous for the structural integrity of the RCDs base were the loads on the anterior blocks of teeth. The stress fields in the areas of their maximum values due to the symmetrical load on the first and second blocks are shown in Figure 8. The stress scale is presented in a normalized form when all the loads are related to the found maximum stress value. The stress isoline step is 10%.

By analyzing the stress fields, it can be concluded that the maximum values were localized in the anterior part of the RCDs base. The distributions of stresses greatly depended on their geometric features; in particular, increased stress levels were found in the vicinity of the technological cutouts for strands.

In the case of loading on Block I, the stress distribution was less uniform in the anterior part of the RCDs base. There was an evident zone located on its axis of symmetry where the stress levels were much lower, despite the high tension of its other part.

Hence, the high-stress gradients took place in the anterior part of the RCDs. The maximum stresses were localized at the tips of the technological cutouts for strands. For this reason, the most probable areas for crack initiation and subsequent fracture were the zones located at its axis of symmetry and at the tips of the technological cutouts for the strands. Under symmetrical loads, the maximum stress level reached 18.8 MPa.

When the load was applied on Block II, the stress distribution differed significantly from that discussed above. The front part of the RCDs base was less stressed. The maximum stresses were localized under the loaded block of teeth and at the tips of the technological cutouts for the strands, which were the most probable regions for crack initiation and subsequent fracture.

**Figure 9:**
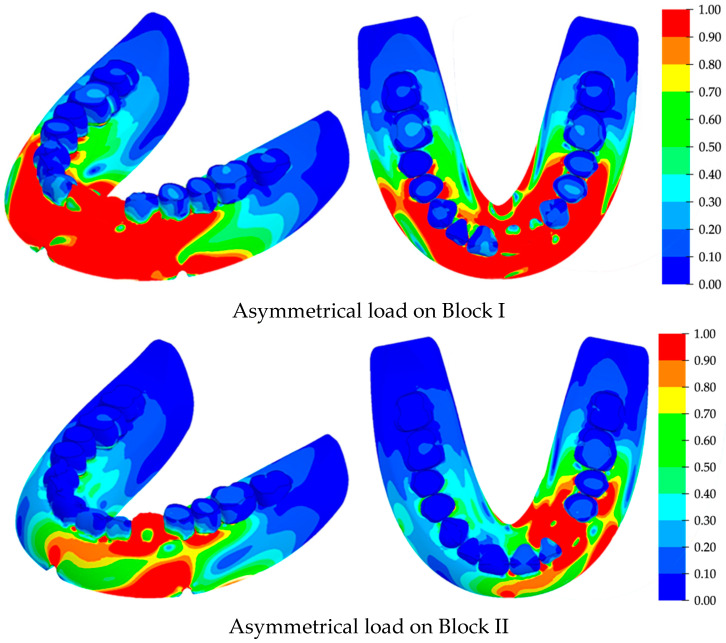
The distributions of the maximum stresses under asymmetrical loads on various blocks of teeth: isometry on the left; the bottom view on the right.

The calculated stress fields under asymmetric loads on Blocks I and II are presented in Figure 9. The key difference between these data and those obtained for symmetrical loading was the greater stress levels, as well as the extension of the increased stress region towards the RCDs base’s back. The regions of maximum local stresses were also located at the anterior part of the denture base, but they were shifted towards the loaded block of teeth. The maximum calculated stresses are presented in Table 2 for various directions and types of applied loads. It could be concluded from their analysis that loading on Blocks I and II, which correspond to the processes of food biting and mastication, are dangerous for the structural integrity of the RCDs base.

Asymmetric loading on Block II caused higher stress levels than those under symmetric loads on Block I. The maximum stresses were localized at the foundation of the tooth blocks, as well as at the tips of the technological cutouts for strands. Significant stress levels were concentrated along the RCDs base up to the positions of the molars. The bottom view shows a high level of stress at the interface between the denture base and the mucosa, which is directly under the projection of the block of teeth. Under asymmetric loads, the maximum stress level was 40.2 MPa. The difference between the stress levels under symmetric and asymmetric loading was 2.2 times. Thereby, the asymmetric loading is more dangerous in terms of prosthesis structural integrity.

### 3.2. Cyclic Loading

In some cases, the fatigue fracture occurs even when RCDs are correctly installed and fitted to the supporting tissues and there is no hard food in a patient’s diet. Already at load levels of about 40% of the ultimate tensile strength of the prosthesis material, a gradual degradation of the latter takes place, resulting in failure. An important practical issue is the prediction of the service life for RCDs under cyclic loads. For this purpose, the concept of material fatigue behavior can be applied with the multi-axial cyclic failure criterion.

Based on the results of both computer simulations and an investigation of cyclic failure, it is possible to establish a characteristic operation period (the number of cycles to a failure) for RCDs. A comparison of the calculated values with corresponding clinical data enables us to understand whether a mistake in the clinical preparation of a patient for prosthetics or a low-fatigue lifetime was the key reason for prosthesis failure. In medicine, a common practice is to replace such RCDs bases after their early failure, but it is not always the right decision. The results of this study provide the correct dental treatment strategy and denture design optimization to prevent such incidents.

For cyclic loading conditions, the concept of a material endurance limit—below which a component does not fail during the specified number of cycles—was employed. In this case, the lifetime depends on the load magnitude and is characterized by a fatigue curve. Such a curve is often considered in semilogarithmic coordinates and is described by a Baskin-type relation [59]:

(1) $$\sigma_{eq}=\sigma_{f}+\sigma_{L}N^{-\beta }$$

It is assumed that at low durability levels (on the order of 10^2^–10^3^ loading cycles), the material experiences significant irreversible strain and failure occurs at stress levels around its yield point. Below this limit, the material is deformed in the separate localized zones, which causes a significant change in its durability with a decrease in the load amplitude.

Assuming that the equivalent stress should be equal to the material yield point at 10^3^ cycles, the following relationship can be written to determine the parameters of the equation:

$$\sigma_{0.2}=\sigma_{f}+\sigma_{L}10^{-3\beta }$$

$$\sigma_{L}=10^{3\beta }\cdot \left(\sigma_{0.2}-\sigma_{f}\right)$$

where $\sigma_{0.2}$ is the yield point, $\sigma_{f}$ is the fatigue limit, and β is an exponent determined via the experimental data. The result is a relationship between the equivalent stress and the number of cycles prior to a failure. In order to denote the key failure mechanisms, one can select different expressions for the equivalent stresses, thereby highlighting the predominant factors that contribute to the fracture.

The SSS was analyzed by FEM-based SolidWorks software. To visualize the durability isolines in terms of the number of the cycles to a failure, the elastic problem was solved, and was obtained with built-in SolidWorks procedures and an additionally developed code that enables us to assess such parameters for each node using a relation similar to Equation (1).

**Figure 10:**
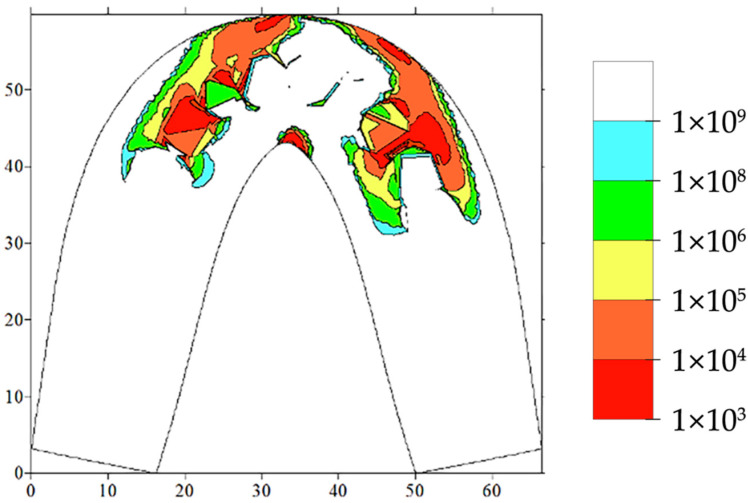
The computer simulation results, presented as isolines of the number of the cycles prior to failure.

An analysis of the numerical results obtained contributed to the conclusion that the most dangerous are the functional loads on the anterior sections of the dentition, namely on the incisors and canines. In this case, the divergence of the back part of the prosthesis base occurred, which indicated the dominant strain mechanism due to the tensile components of the stress tensor (Figure 10). An estimation of the absolute values of these loads allowed us to use the criterion for the multi-axial fatigue failure according to the SSS assessment.

To estimate the number of cycles prior to failure, the classic Smith–Watson–Topler multi-axial criterion was chosen; it considers the cyclic load amplitude, as well as the average stresses due to the RCDs base’s shrinkage in the supporting tissues or residual ones after prosthesis fabrication [60]:

(2) $$\sqrt{\langle \sigma_{1_{\max}}\rangle \Delta \sigma_{1}/2}={\tilde{\sigma }}_{u}+\sigma_{V}N^{-\beta_{VH}}$$

The equivalent stress on the left side of Equation (2) is a combination of the average tensile stress and the cyclic load amplitude. The right side of Equation (2) corresponds to the Baskin fatigue curve and contains three values of the material mechanical characteristics. The *β* exponent determines the downslope rate for the material fatigue curve. Consequently, the left side of Equation (2) completely depends on the prosthesis’s SSS, which is determined via computer simulation results. The right side of Equation (2) includes the material parameters, which are assessed by the fatigue tests (uni-axial symmetric loading), as well as one unknown value, namely the number of cycles prior to failure.

As a result, it was possible to draw durability isolines using the calculated data. An example shown in Figure 10 presents examples for the case of asymmetric loading on the incisors, according to Figure 9. The red color corresponds to the zones where cracking could develop after 1000 cycles. These estimates were performed for the loading conditions, wherein the maximum muscle effort was developed for each cycle. The regions marked in blue and white were considered potentially non-hazardous, since the implementation of more than a billion cycles during the use of the dentures is beyond the average human life expectancy. Thus, the obtained data enabled us to differentiate the regions of maximum equivalent stresses, which are presented in Figure 9, and link the stress values with the number of cycles prior to failure.

**Figure 11:**
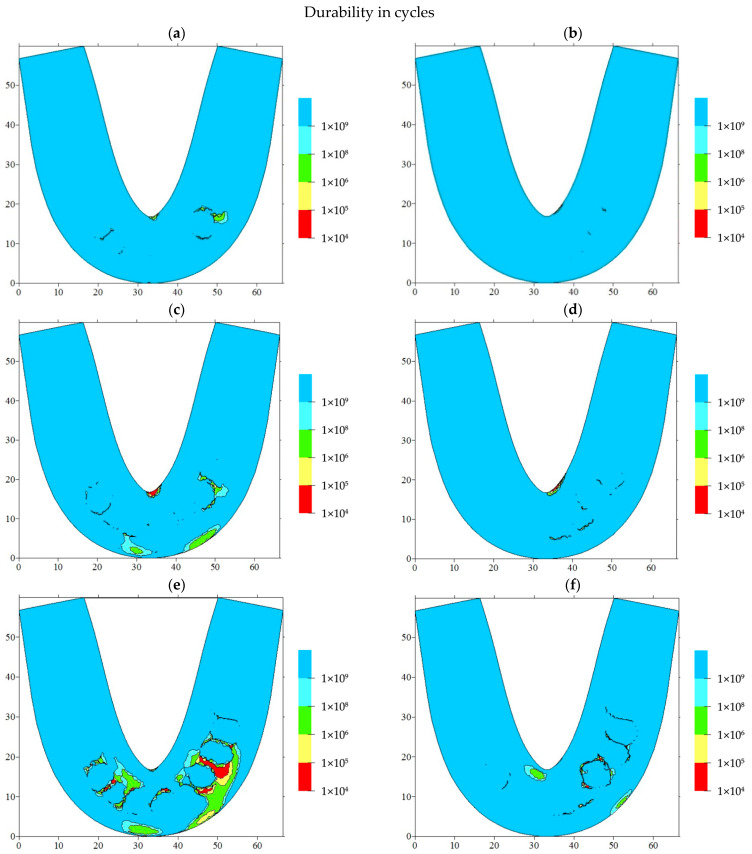
The computer simulation results presented as isolines of the number of the cycles to the failure for various arrangement cases of the blocks of teeth. (**a**) incisors, normal; (**b**) canine, normal; (**c**) incisors, displacement; (**d**) canine, displacement; (**e**) incisors, inclination; (**f**) canine, inclination.

**Figure 12:**
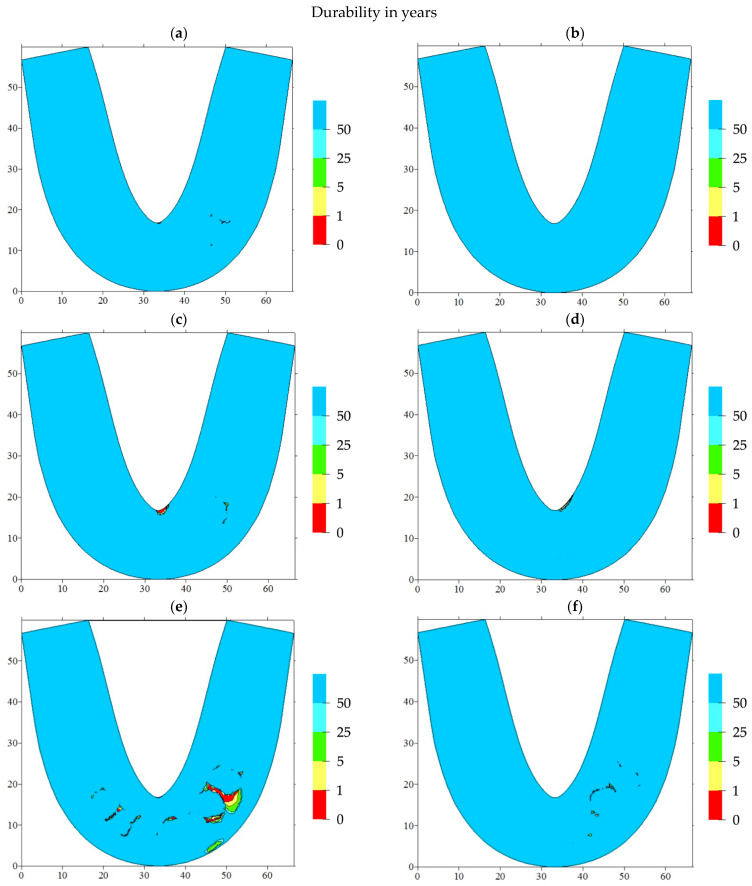
The computer simulation results presented as isolines the service life to the failure (in years) for various arrangement cases of the blocks of teeth. (**a**) incisors, normal; (**b**) canine, normal; (**c**) incisors, displacement; (**d**) canine, displacement; (**e**) incisors, inclination; (**f**) canine, inclination.

Based on the above, it became possible to estimate the service life of such RCDs (in years) by suggesting hypotheses about the frequency of cyclic loads—at which point the maximum mastication force was applied—as well as by analyzing the number of cycles required for thorough food mastication. Figure 11 and Figure 12 show the results of the service life calculations for some of the most dangerous cases, which are expressed in the number of cycles and their corresponding years of operation. 

Loading on the front blocks of teeth caused the formation of potential fracture regions in the front part of the RCDs. Thus, with the normal arrangement of the blocks of incisors, there was such a region located on the denture base axis (Figure 11a).

In addition, the potential fracture regions were caused by transitions from the RCDs base material to the “teeth” one. In this case, the estimated number of cycles prior to failure was about 10^6^. When loads were applied on the block of incisors, another potential fracture area was formed in the anterior part of the dentures base. Respectively, the localization of such regions almost coincided with the case of loading the incisors, but slightly shifted from the denture base axis of symmetry.

In the case of any deviations from the typical arrangement of individual blocks of teeth, a significant decrease in the complete denture service life (by two orders of magnitude) was observed. When the location of the incisors was displaced, one of the potential failure areas dominated, corresponding to the axis of symmetry of the RCDs base (Figure 11c). This area remained the most dangerous in the case of any displacements of canines. Hence, the predominant location of the potential failure area on the axis of symmetry of the denture base was observed with a slight deviation from the recommended arrangement parameters for the artificial teeth.

When the blocks of teeth are tilted, the SSS becomes even more dangerous, as it leads to multi-zone localizations of the potential failure areas. In addition to the characteristic zones on the axis of symmetry, the contact areas between the blocks of teeth and the RCD base are hazardous. If any displacements or tilts take place, the regions of a potential fatigue crack initiation are located both on the oral side of the denture base (the predominant position of the failure initiation region) and on the vestibular one.

Assuming typical cyclic loading conditions for the prostheses as three meals a day, it was possible to present the results in isolines by years of operation. With the normal arrangement of the blocks of artificial teeth, the service life of the denture exceeds a year of operation, and the potential failure regions are localized on the RCDs base axis of symmetry and at the foundation of the blocks of the front teeth. In the case of deviations from the recommended standards, a fracture may occur during the first year of operation. At the same time, any displacements of the teeth from the occlusal scheme could give rise to the localization of potential failure areas on the RCDs base axis. Any inclinations that contribute to their multi-zone origination and localization are mainly at the base of the blocks of anterior teeth.

## 4. Discussion

The obtained results can be used to interpret complex failure cases, especially when the regions of the fatigue crack initiation are observed at “atypical” locations. A promising study is the development of an evolutionary model of crack propagation, which took into account the material’s damageability. In this case, it is possible to obtain some new data about the relationship between the crack propagation trajectory and the loading mode, as well as the occlusal schemes for individual blocks of teeth.

According to the goal and the objectives that the authors stated at the beginning, they solved, first of all, the issue of developing an integrated approach for the treatment of edentulous patients. For this reason, a simplified approach based on the SolidWorks package was used for FE modeling. Since this paper describes the results as being obtained in the first stage of research, it possesses several limitations. Among them are: the mesh division details were poorly described; the convergence test was not presented; the information about mesh density and boundary conditions was hardly reproducible; and the anatomy was simplified and the contact point was not representative. However, in forthcoming studies, all these shortcomings shall be eliminated.

One more weak point of the described results was the lack of validation of the FEM methodology [50], and this can be explained by several reasons: (1) the authors are currently studying various dental materials for manufacturing products by 3D-printing and this work is not completed; (2) the development of the final digital technology for manufacturing RCDs is also not completed; and (3) the purpose of this research was not to verify the FE model, but rather to develop a computing base that allows dentists to promptly solve problems and adjust their treatment strategies.

Despite the above, the authors clearly understand the importance of the problem relating to FE model verification. The research dealing with the experiments on the cyclic loading of dental material and RCDs are underway and the authors have every intention of informing the scientific community about the results in forthcoming publications.

**Figure 13:**
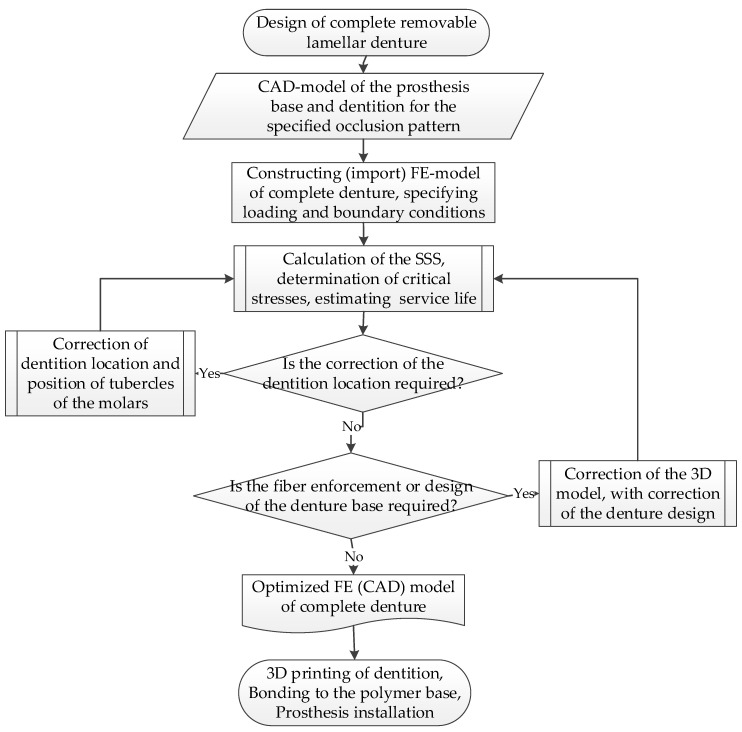
The algorithm for designing RCDs, including the stage of the FEM analysis of its service life.

On the state formulation, the FEM analysis becomes not just a way to simulate the deformation behavior of the denture structures, but also becomes a part of the occlusal scheme design for the treatment of edentulous patients. This enables us to flexibly solve the issue of prolonging the service life of RCDs by varying the arrangement of the artificial teeth (simulating options for occlusion of the dentition) on the base. The latter, in turn, is in contact with the supporting tissues. According to the numerical simulation results, not only can the service life of the dentures be prolonged, but also the load transferal from the RCDs to the supporting tissues can be reduced. An algorithm for designing RCDs, including the stage of FEM analysis of its service life, is presented in Figure 13.

It should be noted that the FEM analysis enabled us to determine the optimal variant of occlusion according to the calculated SSS pattern by means of a virtual change in the position of functionally oriented groups of teeth in the anteroposterior (vestibulo-oral) direction (or individual teeth in these groups not only in the vestibulo-oral direction, but also around their axis). In particular, it is possible to assess the most correct location of the cusps of molars in relation to ones from opposing teeth (which can change the occlusal scheme). As a result, with the same force developed by the masticatory muscles, a variation in the contact area is accompanied by a change in the local pressure by several times. This causes multiple decreases in the load that is transferred to the RCDs base.

Incorrect (and even irrational) occlusion-oriented (adapted) opposing dentitions affect the stability of the dentures on the supporting tissues (the edentulous alveolar ridge). Given this, the dentures topple over in the functioning process, in particular when talking. This is accompanied by a lack of stabilization (fixation). At the same time, the dentures can be held extremely firmly on a jaw out of the occlusal contact, thereby providing a high retention level.

**Figure 14:**
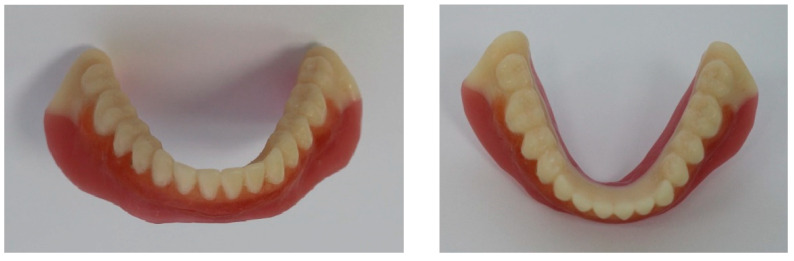
Mandibular RCDs, Digital Light Processing (DLP) printed using a digital model imported from SolidWorks software package.

Another advantage of designing a dental prosthesis in a CAD system is rapid prototyping [61,62]. Based on the results of the correction of the artificial teeth, it was manufactured using a 3D printer. The printed blank can be quickly bonded to the RCDs base (which can also be fabricated by additive manufacturing) with a polymer adhesive. Along with this, removable dentures can be printed monolithically in accordance with the dentist’s specifications. Firstly, the dentition can be 3D-printed; then, an operator changes a feedstock and prints a pink color plastic base (Figure 14). Upon completion of 3D-printing and post-polymerization processing, the entire vestibular surface is finalized and individualized by color and surface roughness (Figure 14).

Thus, the virtual complete denture fitting minimizes the risk of occlusal disorders, which can contribute to the improved retention and stabilization of RCDs during their usage.

Due to the individuality of each patient, when manufacturing RCDs for him/her, predicting the duration of its operation is extremely difficult. Therefore, the purpose of this research was to assess the effect of the location of the artificial teeth on a foundation on the service life of RCDs; however, the goal of the authors, as representatives of the dental community, was not to develop a fundamentally new FEM-based model for calculating the service life of complete dentures using self-designed software.

An example of RCDs fabricated by 3D-printing, which took into account the FEM analysis results, is shown in Figure 14. These studies are currently being introduced into the practice of dental treatment at the 3rd State Medical University (Moscow, RF).

## 5. Conclusions

An algorithm for designing complete RCDs, including the stage of FEM analysis of its service life, was proposed. In this case, the FEM analysis was conducted with commercial software packages (a “black box” with a minimum possibility of varying the model parameters) and became not just a way to simulate the deformation behavior of the dentures’ structures, but also became a part of the treatment of edentulous patients. This enabled us to flexibly solve the issue of prolonging the service life of RCDs by varying the arrangement of the artificial teeth (simulating options for occlusion of the dentition) on the base. According to the numerical simulation results, the load transmitted from the complete dentures to the supporting tissues could be reduced.

The complex models of the RCDs base were proposed for a mandible, which considered the characteristic features of the anatomical structure of the mucous membrane of the denture base. The static and cyclic strength of the dentures were investigated for various arrangement cases of individual blocks of teeth. The number of cycles prior to a failure was estimated, and the localized fatigue failure zones were revealed as well. The model can be adopted for the FEM simulation of a maxilla.

It was shown that both the displacement and inclination of individual blocks of teeth exert a negative impact on the cyclic durability of the prostheses. When the occlusal scheme of the front blocks is shifted, their durability can be reduced by two orders of magnitude, which is in contrast with the case for a typical occlusal scheme. The displacement of the arrangement line of individual blocks of teeth caused potential failure area localization on the RCDs base axis of symmetry. In this case, the nucleation of a crack could take place on both the interior and vestibular sides of the dentures. Any inclinations of the blocks of teeth resulted in a complex spatial distribution of the stress fields and fractures through the foundation of the blocks of artificial teeth.

The obtained results enable us to interpret the clinical fracture cases, as well as to determine the causes of “non-typical” fatigue failure locations.

## Figures and Tables

**Table 1:** The mechanical properties of the acrylic plastics used for the denture prosthesis. Reproduced with permission from [Sergey D. Arutyunov, Dmitry I. Grachev, Grigoriy G. Bagdasaryan et al.], [Numerical Study on the Teeth Installation Parameters: Shift and Tilt Angle Effects]; published by [Springer], [2021].

Material	Application	Mechanical Properties
Acrylic plastic R	Denture base	Young’s modulus 1000 Mpa Density 1000 kg/m^3^ Poisson ratio 0.3
Acrylic plastic W	Teeth	Young’s modulus 2000 MPa Density 1000 kg/m^3^ Poisson ratio 0.3

**Table 2:** Maximum stress levels for different loaded blocks of teeth.

Load	Block I	Block II	Block III	Block IV
Symmetric, MPa	18.8	14.4	2.93	1.1
Asymmetric, MPa	40.2	22.14	5.67	2.34

## Data Availability

The data presented in this study are available on request from the corresponding author.
